# Frequency Responses of Rat Retinal Ganglion Cells

**DOI:** 10.1371/journal.pone.0157676

**Published:** 2016-06-24

**Authors:** Alex E. Hadjinicolaou, Shaun L. Cloherty, Yu-Shan Hung, Tatiana Kameneva, Michael R. Ibbotson

**Affiliations:** 1 National Vision Research Institute, Australian College of Optometry, Carlton, Victoria, Australia; 2 ARC Centre of Excellence for Integrative Brain Function and Department of Optometry and Vision Sciences, University of Melbourne, Parkville, Victoria, Australia; 3 Department of Electrical and Electronic Engineering, University of Melbourne, Parkville, Victoria, Australia; Instituto Murciano de Investigación Biosanitaria-Virgen de la Arrixaca, SPAIN

## Abstract

There are 15–20 different types of retinal ganglion cells (RGC) in the mammalian retina, each encoding different aspects of the visual scene. The mechanism by which post-synaptic signals from the retinal network generate spikes is determined by each cell’s intrinsic electrical properties. Here we investigate the frequency responses of morphologically identified rat RGCs using intracellular injection of sinusoidal current waveforms, to assess their intrinsic capabilities with minimal contributions from the retinal network. Recorded cells were classified according to their morphological characteristics (A, B, C or D-type) and their stratification (inner (i), outer (o) or bistratified) in the inner plexiform layer (IPL). Most cell types had low- or band-pass frequency responses. A2, C1 and C4o cells were band-pass with peaks of 15–30 Hz and low-pass cutoffs above 56 Hz (A2 cells) and ~42 Hz (C1 and C4o cells). A1 and C2i/o cells were low-pass with peaks of 10–15 Hz (cutoffs 19–25 Hz). Bistratified D1 and D2 cells were also low-pass with peaks of 5–10 Hz (cutoffs ~16 Hz). The least responsive cells were the B2 and C3 types (peaks: 2–5 Hz, cutoffs: 8–11 Hz). We found no difference between cells stratifying in the inner and outer IPL (i.e., ON and OFF cells) or between cells with large and small somas or dendritic fields. Intrinsic physiological properties (input resistance, spike width and sag) had little impact on frequency response at low frequencies, but account for 30–40% of response variability at frequencies >30 Hz.

## Introduction

The rat has proven to be a useful model for investigating the visual system (e.g. [1]), including visual dysfunction (e.g. [2]). However, the rat retina is not as well characterized as others and knowledge about the physiology of the retinal ganglion cells (RGCs) is an important consideration [3–5]. RGCs in rats and other mammals are tasked with encoding visual information into electrical signals and conveying those signals to the brain via the optic nerve. These cells take a variety of anatomical forms distinguished by soma size, dendritic field size, branching patterns, and stratification [6–8]. These morphological characteristics, together with the distribution of ion channels on the cellular membrane, give rise to intrinsic properties that influence the encoding of visual information [9,10].

RGCs vary in their response to light stimulation [8]. Heine and Passaglea [4] showed that most rat RGCs have response properties similar to the well-characterised cat X- and Y-cells, i.e. brisk responses, center-surround receptive fields (RFs), and linear or nonlinear spatial summation. Other rat RGCs had response properties similar to various types of mammalian W-cells, e.g. local-edge-detectors and suppressed-by-contrast cells. Many RGC types can be divided into ON and OFF subtypes. ON cells respond to luminance increments within their receptive field while OFF cells respond to luminance decrements. ON-OFF cells respond to both increments and decrements. These functional signatures are determined by the cells’ dendritic stratification—ON cells stratify within sublamina a of the inner plexiform layer, OFF cells stratify in sublamina b, and ON-OFF cells stratify in both sublaminae [7,10]. Moreover, ON and OFF retinal pathways exhibit a number of other functional differences, including receptive field size, contrast adaptation and contrast sensitivity [11,12].

Wong et al. [5] conducted an extensive survey of the intrinsic electrical properties of 16 rat RGC types. This survey assessed responses to step changes in injected current but did not evaluate the influence of the frequency at which the current is injected. Here we add to the growing knowledge of rat RGC physiology by investigating the frequency responses of RGC by injecting sinusoidal currents at various frequencies, simulating the synaptic input resulting from sinusoidally modulated luminance changes [13]. Previous efforts to characterise the temporal frequency tuning of RGCs have involved visual grating stimuli (e.g. [14]), which engage the entire retinal network as per regular physiology. In our study we make use of intracellular current stimuli in a bid to minimize the influence of the network on the cell’s innate frequency response. Our primary aim was to assess the extent to which RGC frequency responses are related to morphological classification, cell size or dendritic stratification. Further, the responses of RGCs to repetitive stimuli are known to decay with both time and stimulus frequency (e.g., [15]). Adaptation is a widespread phenomenon in the visual system that is likely to involve both intrinsic and pre-synaptic mechanisms [16–18]. Another aim of our study was to investigate the role of intrinsic mechanisms in adaptation, by measuring the decay of responses over time and determining whether this decay depends on cell type.

## Materials and Methods

All experimental procedures and reagents used for this study were approved by the Faculty of Science Animal Ethics Committee, at the University of Melbourne.

### Retinal whole-mount preparation

Whole-cell current clamp recordings from 150 RGCs were obtained using procedures described previously [5,9]. Retinae were explanted from 56 Long Evans rats that were aged between 6 and 11 months and sourced from Monash Animal Services. Animals were anaesthetised by intraperitoneal injection of a mixture of Ketamine (100 mg/kg) and Xylazine (10 mg/kg). Once deeply anaesthetized, both eyes were enucleated, after which the animal was immediately killed by intracardiac injection of an overdose of Sodium Pentobarbital (350 mg). Quadrants of retinal whole-mounts were placed, ganglion cell layer up, in a recording chamber (Warner Instruments, Hamden, CT USA, RC-26 GLP) and perfused (4–6 ml/min) with carbogenated Ames' medium (Sigma-Aldrich, St. Louis, MO) at room temperature. The chamber was mounted on the fixed stage of an upright microscope (Olympus, BX51WI) fitted with a 40× water immersion lens. Tissue was visualised on a monitor with 4× additional magnification.

### Physiological data collection

Before a whole-cell recording of a ganglion cell was possible, access to the cell surface was obtained by making a small hole in the inner limiting membrane and optic fiber layer overlying the cell [5,9,12,19]. Recordings were limited to RGCs exposed during this procedure that had smooth surfaces and agranular cytoplasm. The pipette internal solution contained (in mM): K-gluconate 115, KCl 5, EGTA 5, HEPES 10, Na-ATP 2, Na-GTP 0.25; (mOsm = 273, pH = 7.3) including Alexa Hydrazide 488 (250 μM) and biocytin (0.5%).

Whole-cell current clamp recordings from RGCs were obtained with standard procedures [20]. Initial pipette resistance was 3–7 MΩ. After measurement, this resistance was compensated using the bridge balance circuit of the amplifier. Resting potentials were corrected for the change in liquid junction potential that develops upon break-in and cell dialysis, measured directly as -5 mV [21]. No capacitance compensation was employed to avoid inducing unwanted oscillations in the recordings, which can lead to loss of the high-resistance seal formed between the pipette and the soma.

The membrane potential was amplified (BA-1S, NPI), digitized with 16-bit precision at a rate of 20 kHz (USB-6221, National Instruments) and stored in digital form. Experiment control and data acquisition were performed using custom software developed in LabVIEW (National Instruments). Cells were excluded from analysis if they exhibited markedly inconsistent responses to repeated presentations of the test stimuli, or if their morphological classification could not be reliably ascertained after performing the immunocytochemistry.

### Electrical stimulation

Electrical stimuli consisting of sinusoidal current waveforms ranging in frequency from 1–60 Hz (1, 2, 5, 10, 15, 20, 25, 30, 45, 60 Hz) were delivered intracellularly via the recording pipette. The current amplitude was selected so as to compensate for the input resistance of each cell, which can vary over three orders of magnitude among different rat RGC types [5]. Specifically, we injected a sinusoidal current of 10 Hz and varied the amplitude to identify the smallest current that evoked at least one spike in each stimulus cycle. This current amplitude was then used to test all other frequencies. In a majority of cells (107/150), multiple spikes were evoked in at least one stimulus cycle, leading to a spiking frequency of greater than 10 Hz for the given stimulus amplitude.

A single trial consisted of intracellular injection of the sinusoidal current of a given frequency for a period of 1 second. The response of the cell was quantified as the number of evoked spikes over this 1 s period, and labelled as *spiking frequency*. An individual neuron’s frequency response was then defined as the cell’s spiking frequency as a function of stimulus frequency. Each stimulus frequency was trialed at least twice, with an average number of 2.8 trials collected for each stimulus condition.

In addition to the sinusoidal test currents, each cell was also subjected to 400 ms rectangular pulses of both hyperpolarizing and depolarizing current. Recordings of the membrane potential during these current pulses was used to quantify the intrinsic properties of each recorded cell [5].

### Histochemistry and morphological classification

After recordings, the tissue was removed from the chamber, mounted onto filter paper, fixed for approximately 45 minutes in phosphate-buffered 4% paraformaldehyde, and stored for up to 2 weeks in 0.1 M phosphate-buffered saline (PBS; pH 7.4) at 4°C. Subsequent processing of the tissue revealed biocytin-filled cells by immersion in PBS with 0.5% Triton X-100 and 20 g/mL streptavidin conjugated to Alexa488 (Invitrogen) overnight. Tissue was thoroughly washed in PBS and stained with propidium iodide for roughly 8 minutes revealing the boundaries of the inner plexiform layer (IPL) by staining the nuclei of cells in the inner nuclear (INL) and ganglion cell layers (GCL). After additional washes in PBS, samples were mounted onto Superfrost slides and protected in 60% glycerol using a coverslip.

**Fig 1:**
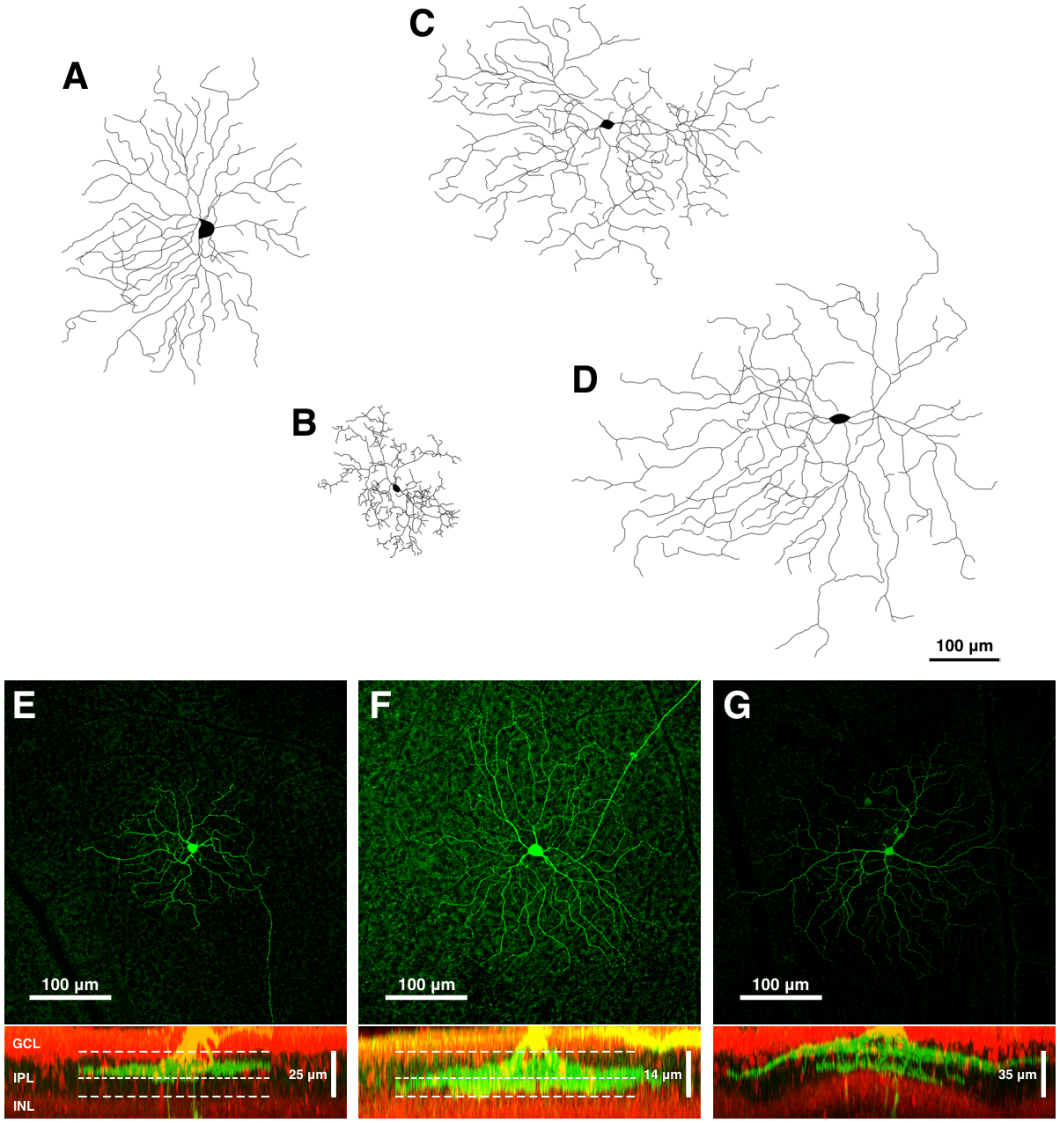
Reconstruction of recorded cell morphology. Representative confocal image stacks typical of those used for classification of recorded retinal ganglion cells (RGCs) according to their morphological cell type. Examples are shown for representative (A) A-type, (B) B-type, (C) C-type, (D) D-type, (E) ON [C2i], (F) OFF [A2o], and (G) ON-OFF [D2] RGCs. Panels (E–G) show the *en face* representation (top), revealing the scale and extent of the dendritic arborisation, and a cross-section (bottom), revealing where the dendrites of each cell stratify within the inner plexiform layer (IPL). Long-dashed lines indicate the boundary of the IPL and short-dashed lines indicate the approximate boundary between the two sublaminae. Recorded cells were labelled, via the patch pipette, with Alexa488 (green). Other cells in the ganglion cell layer (GCL) and inner nuclear layer (INL) were labelled with propidium iodide (red).

Ganglion cells were reconstructed in 3D with a confocal microscope (Zeiss PASCAL) and classified morphologically into types according to well-established criteria [5,7,10]. Reconstructions for 100 of the 150 recorded cells were collected—cells that could not be classified were excluded from further analysis. Generally, cells classified as A-type (A1, A2; n = 43) had large somata and large dendritic fields (Fig 1A and 1F), cells with small-to-medium-sized somata and dendritic fields were classified as B-type (B2, B3, B4; n = 4) (Fig 1B), and cells with small-to-medium-sized somata and medium-to-large dendritic fields were designated as C-type (C1, C2, C3, C4; n = 27) (Fig 1C and 1E). Bistratified cells were classified as D-type RGCs D1, D2; n = 26) (Fig 1D and 1G).

Cells were further classified on the basis of their dendritic stratification (*s*), quantified as a percentage of the inner plexiform layer (IPL) thickness, according to,

$$s\left(x\right)=100\left(1-\frac{IPL_{s}-x}{IPL_{s}-IPL_{e}}\right)$$ (1)

where *x* refers to the depth of a terminal dendrite, and *IPL*_*s*_ and *IPL*_*e*_ refer to the depth of the start and end of the inner plexiform layer, respectively. Here, depth increases from the ganglion cell layer towards the photoreceptor layer. Cells that stratified in the inner part of the IPL (*s* ≥ 45%), nominally ON cells, are denoted throughout by an ‘i’, e.g. a C4i cell is an ON cell of the C4 cell type. Classification of cells as ON-type was relatively simple as they were cells that do not send dendrites below the threshold level (Fig 1E). However, categorical classification of OFF-cells was far more difficult in the rat because in most cases these cells had the majority of their dendrites in the outer part of the IPL (e.g. *s* ≤ 35%) but it was often the case that some dendrites were present also in the inner IPL. However, dendrites in the inner IPL were not in the form of a segregated dendritic layer (Fig 1F). Rather, putative OFF-cells have some dendrites in the inner IPL for the simple reason that the dendrites reached across the inner IPL from their somas into the outer IPL. Thus, the classification of nominal OFF cells, as denoted by an ‘o’ (e.g. C4o) must be regarded with caution. Cells classified as ON-OFF types were all bistratified (e.g. only D-type cells) in our study and had to have two clearly defined and segregated dendritic layers, one in the inner and the other in the outer layer (Fig 1G). Thus, while ON-cell and ON-OFF-cell classification was robust, OFF-cell classification was less categorical based on morphology alone.

### Data analysis

All data analysis was performed off-line using custom software developed in MATLAB (MathWorks). To explore the effect of cell size on spiking frequency, two subsets of cells (large and small) were identified on the basis of soma size and dendritic field size for the A2, C2, D1 and D2 subtypes, where n > 8. Statistical comparisons were performed using two-sample t-tests. Unless otherwise stated, differences were deemed significant for p < 0.05.

## Results

We made *in vitro* whole-cell patch-clamp recordings from 150 RGCs in whole-mount preparations of explanted rat retina. Of these, 50 cells could not be reliably classified on the basis of their reconstructed morphology and were therefore omitted from the analysis presented here. For the remaining cells we compare frequency responses across the different morphological RGC types (A-, B-, C-, and D-type cells), dendritic stratification depths (ON and OFF cells) and on the basis of cell size (large and small cells).

### Morphological RGC types

**Fig 2:**
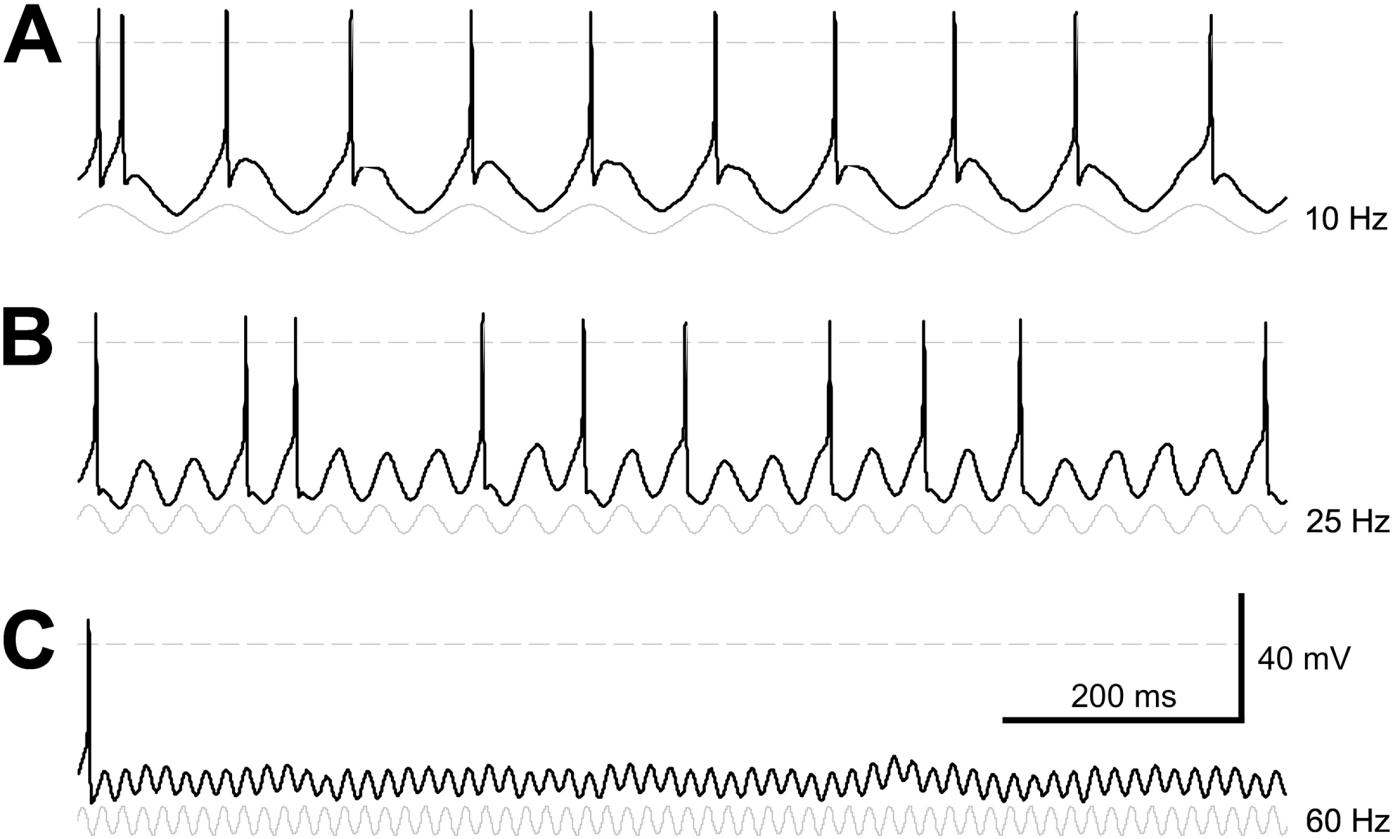
Patch-clamp recording of responses to sinusoidal stimulus currents. Representative membrane potential recordings from an A2o-type retinal ganglion cell, during intracellular injection of sinusoidal stimulation currents (70 pA) at (A) 10 Hz, (B) 25 Hz, and (C) 60 Hz. The frequency and phase of the injected currents are indicated by the sinusoids (gray) shown below each membrane potential recording (black). The dashed line indicates 0 mV.

**Fig 3:**
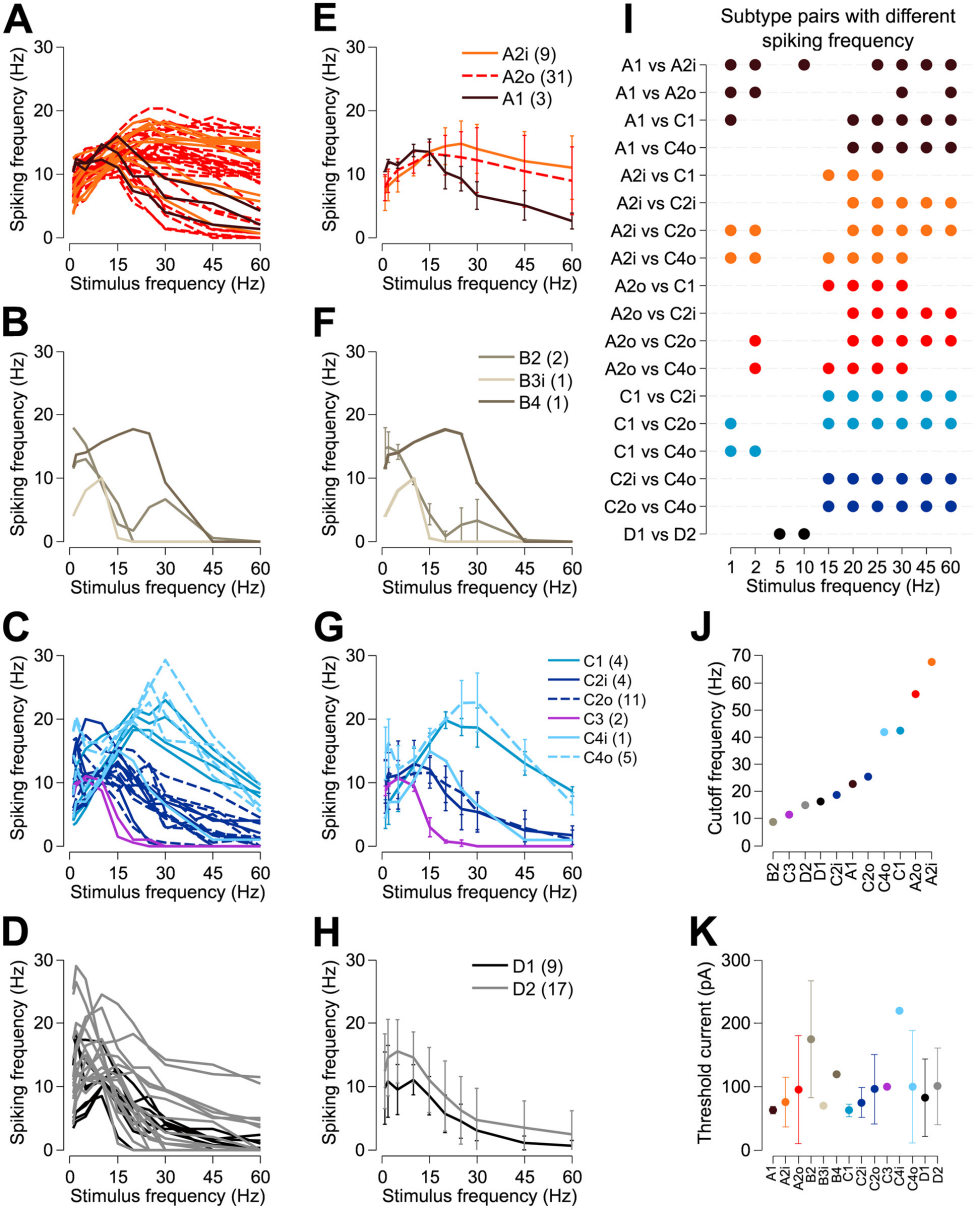
Frequency response of retinal ganglion cells. Panels (A–D) show the recorded frequency response of each recorded cell grouped by morphological cell type (A, A-type; B, B-type; C, C-type and D, D-type). Panels (E–H) show the frequency response of each morphological cell type, averaged across all recorded cells of each type. Error bars indicate plus/minus one standard deviation. These data are summarized in panel (J), which shows the 3 dB cut-off frequency for each morphological cell type for which at least two cells were recorded. Panel (I) summarizes the significant differences between morphological cell types at each stimulation frequency (t-tests, p < 0.05). (K) Mean threshold currents for 10 Hz sinusoidal stimulation for each cell type. Error bars indicate plus/minus one standard deviation. The cell counts for each morphological cell type are indicated in parentheses in the figure legends.

Fig 2 shows patch-clamp recording of responses to intracellular injection of sinusoidal stimulus currents in an A2o-type RGC at 10 Hz (A), 25 Hz (B), and 60 Hz (C). It is evident for this cell that a membrane oscillation occurs for every cycle for all three frequencies. However, only for 10 Hz is a spike also generated by every cycle of the input stimulus. We found significant differences in the frequency spike-responses of different morphological RGC types (Fig 3). The frequency responses of each recorded cell, grouped by morphological cell type, are shown in Fig 3A–3D. For each cell, spiking frequency over the test period was averaged across trials for each stimulus frequency tested. Fig 3E–3H shows the mean frequency response, averaged over all cells, for each morphological cell type. We compared spiking frequency between cell subtypes within each broad morphological cell type (A-, B-, C-, and D-type), with significant differences at each stimulus frequency summarized in Fig 3I. Threshold currents (i.e., the minimum current amplitude required to elicit at least one spike per stimulus cycle for a 10 Hz sinusoid) for each subtype are shown in Fig 3K.

**Fig 4:**
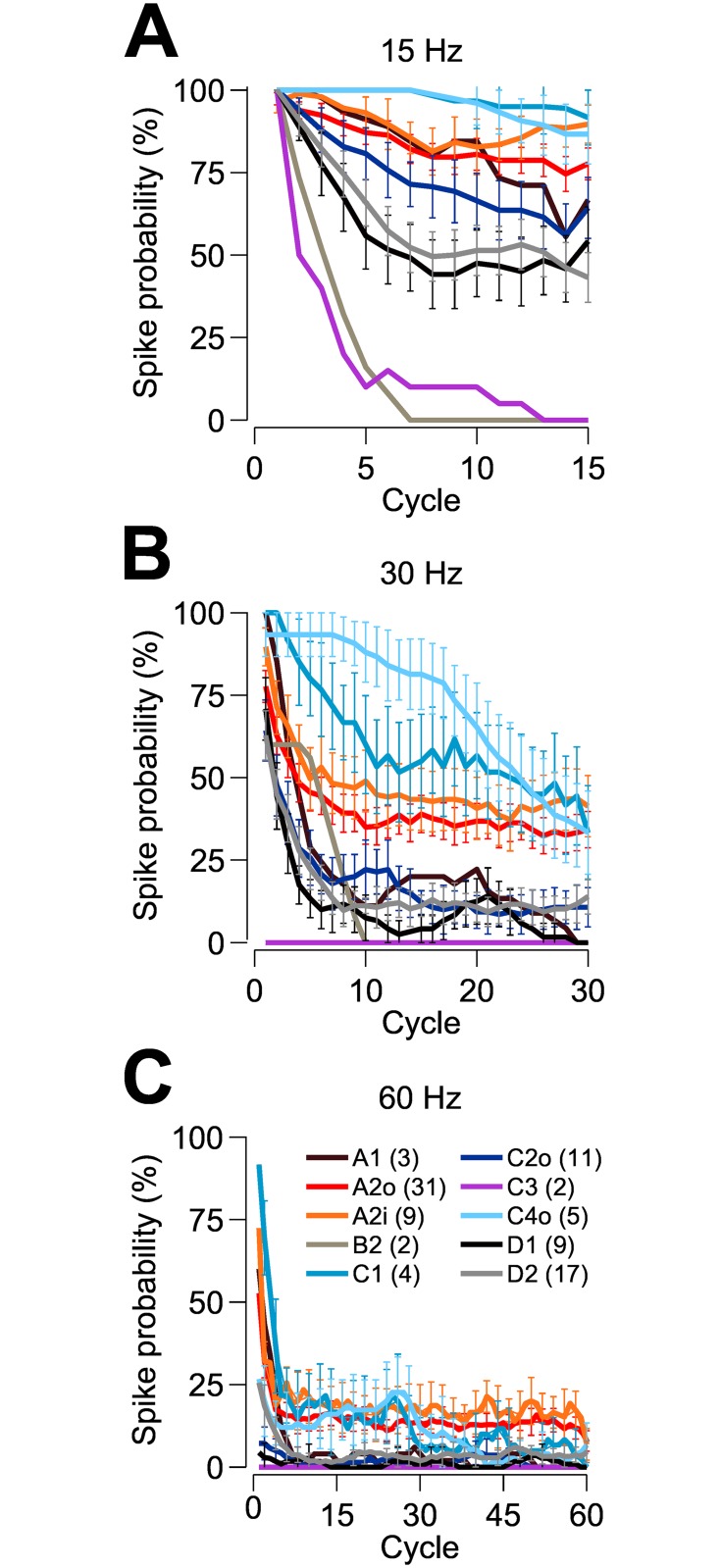
Temporal responses to sinusoidal stimulus currents. (A–C) Probability of observing a spike plotted against time (i.e., cycle count) within each stimulus presentation for stimulus frequencies of 15 Hz, 30 Hz and 60 Hz, respectively. Spike probability curves have been smoothed with a five-point moving average. Error bars indicating plus/minus one standard error have been included for cell types with n ≥ 4. Retinal ganglion cells exhibit a progressive decline in their ability to respond to the depolarizing phase of the stimulus over the course of the trial. This decline is more dramatic for some morphological cell types than for others, and becomes more pronounced as the stimulus frequency is increased.

While most cells exhibited either low-pass or band-pass frequency responses, some cells (particularly within the A2 class) exhibited high-pass responses over the range of frequencies tested (see Fig 3A). The 3 dB cut-off frequency for the mean response of each subtype is shown in Fig 3J. The A2i and A2o cell types are most responsive to high frequency stimulation and have the greatest bandwidth, with 3 dB cut-off frequencies of 68 Hz and 56 Hz, respectively. The spiking frequency of these cells plateaued to a rate greater than that of every other cell type for 60 Hz stimuli, responding to ~17% of stimulus cycles at this frequency (Fig 4C). Notably, the C1 and C4o cell types maintain their response to sinusoidal stimulation more reliably than any other cell type within the 15–30 Hz frequency band (Fig 3I; also see Fig 4A and 4B).

### Dendritic stratification depth

**Fig 5:**
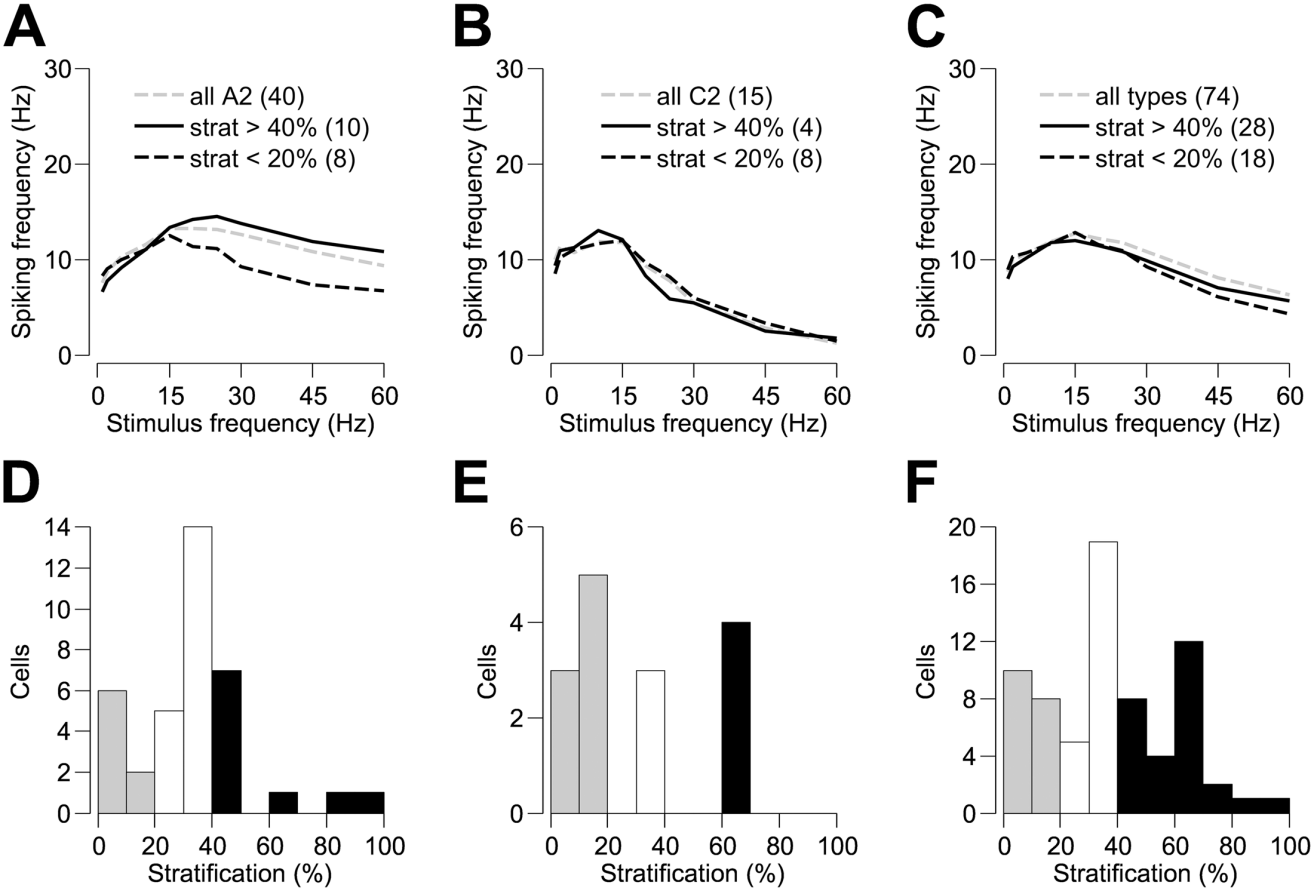
The effect of dendritic stratification depth on the frequency response of retinal ganglion cells. Panels (A) and (B) show the mean frequency response of A2 and C2 RGC types, respectively. Within each RGC type, cells are grouped and their frequency responses averaged according to the depth of their stratification within the inner plexiform layer (IPL). Cells stratifying in the inner-most sub-lamina (>40% of the IPL thickness) are shown in black and those at the outer-most extreme of the IPL (<20% of the IPL thickness) are shown in grey. For comparison, the frequency response, averaged over all cells of a given type, irrespective of stratification depth, is shown by the dashed line. Panel (C) shows the mean frequency response over all non-bistratified RGCs in our sample, grouped according to the depth of their stratification within the IPL but without regard to their morphological cell type. Conventions are the same as in (A) and (B). Panels (D–F) show distributions of dendritic stratification depth, as a proportion of IPL thickness, for the cell groups shown in panels (A–C), respectively.

To investigate the effect of dendritic stratification depth on the frequency response we used data from the two most common cell types in our sample, the A2 and C2 cells. Both cell types stratify at a range of depths within the inner plexiform layer. To assess the effect of stratification depth we compared the frequency responses, within each cell type (either A2 or C2), of those cells stratifying in the inner-most sub-lamina (>40% of the IPL thickness) with those cells having the majority of their dendrites towards the outer-most extreme of the IPL (<20% of the IPL thickness). Fig 5A and 5B show mean frequency responses for these two restricted cell groups within the A2 and C2 cell types, respectively. Distributions of dendritic stratification depth, as a proportion of IPL thickness (grey bars designating <20% stratification; black bars >40% stratification), are shown in Fig 5D and 5E for all A2 and C2 cells, and in Fig 5F for all cell types recorded from in our sample. Among the A2 cells, those stratifying in the inner-most sub-lamina of the IPL were on average more responsive to higher stimulus frequencies than those stratifying closer to the outer limit of the IPL. This difference was very close to the established statistical significance level of p = 0.05 (p = 0.053). We found no significant effect of stratification depth on frequency response among our sample of C2 cells (p > 0.362; Fig 5B). We repeated this analysis on our entire sample of non-bistratified cells (pooling cells of all morphological types) and again found no significant difference between ON and OFF cell frequency responses (p > 0.445; Fig 5C).

### Soma and dendritic field size

We now explore the effect of soma and dendritic field size on the observed frequency response of A2, C2, D1 and D2 cell types. We recorded too few of each of the remaining morphological cell types to perform a meaningful analysis. Since we observe little difference between ON and OFF cell responses within A2 and C2 cells (Fig 5), and find very similar distributions in soma diameter (A2i: 22.2 ± 2.8 μm versus A2o: 21.3 ± 2.2 μm; C2i: 15.5 ± 0.6 μm versus C2o: 15.8 ± 1.9 μm) and dendritic field diameter (A2i: 391 ± 47 μm versus A2o: 394 ± 53 μm; C2i: 315 ± 78 μm versus C2o: 335 ± 53 μm), we combine inner and outer groups for this analysis.

**Fig 6:**
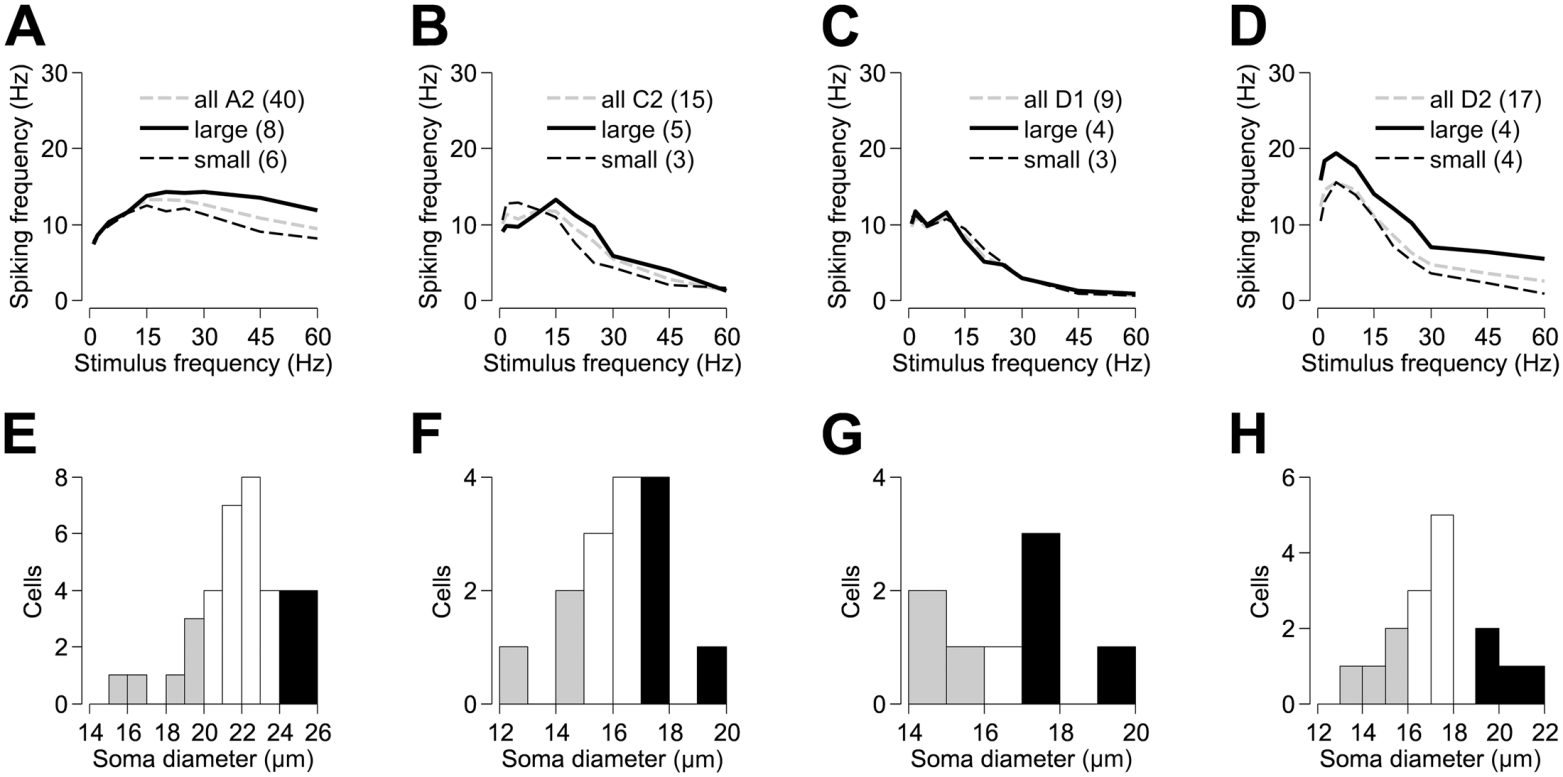
The effect of soma size on the frequency response of retinal ganglion cells. Panels (A–D) show the mean frequency response of A2, C2, D1 and D2 RGC types, respectively. Within each RGC type, cells are grouped and their frequency responses averaged according to their soma diameter. Within each RGC type, cells with the largest soma diameters are shown in black and those with the smallest soma diameters are shown in grey. For comparison, the frequency response, averaged over all cells of a given type, irrespective of soma diameter, is shown by the dashed line. Panels (E–H) show distributions of soma diameter for each of the A2, C2, D1 and D2 RGC types, respectively.

Fig 6A–6D show frequency responses for cells with large and small soma diameters within each morphological cell type. Fig 6E–6H show distributions of soma diameter for each cell type and indicate the upper and lower tails of each distribution designated as large (black bars; {A2, C2, D1, D2 soma diameter} ≥ {24, 17, 17, 19 μm}) and small (grey bars; {A2, C2, D1, D2 soma diameter} ≤ {20, 15, 16, 16 μm}) cells, respectively. For each morphological cell type, cells with larger soma diameters were not significantly more responsive to stimuli at frequencies ≥ 20 Hz (A2: p > 0.159; C2: p > 0.109; D1: p > 0.584; D2: p > 0.244).

**Fig 7:**
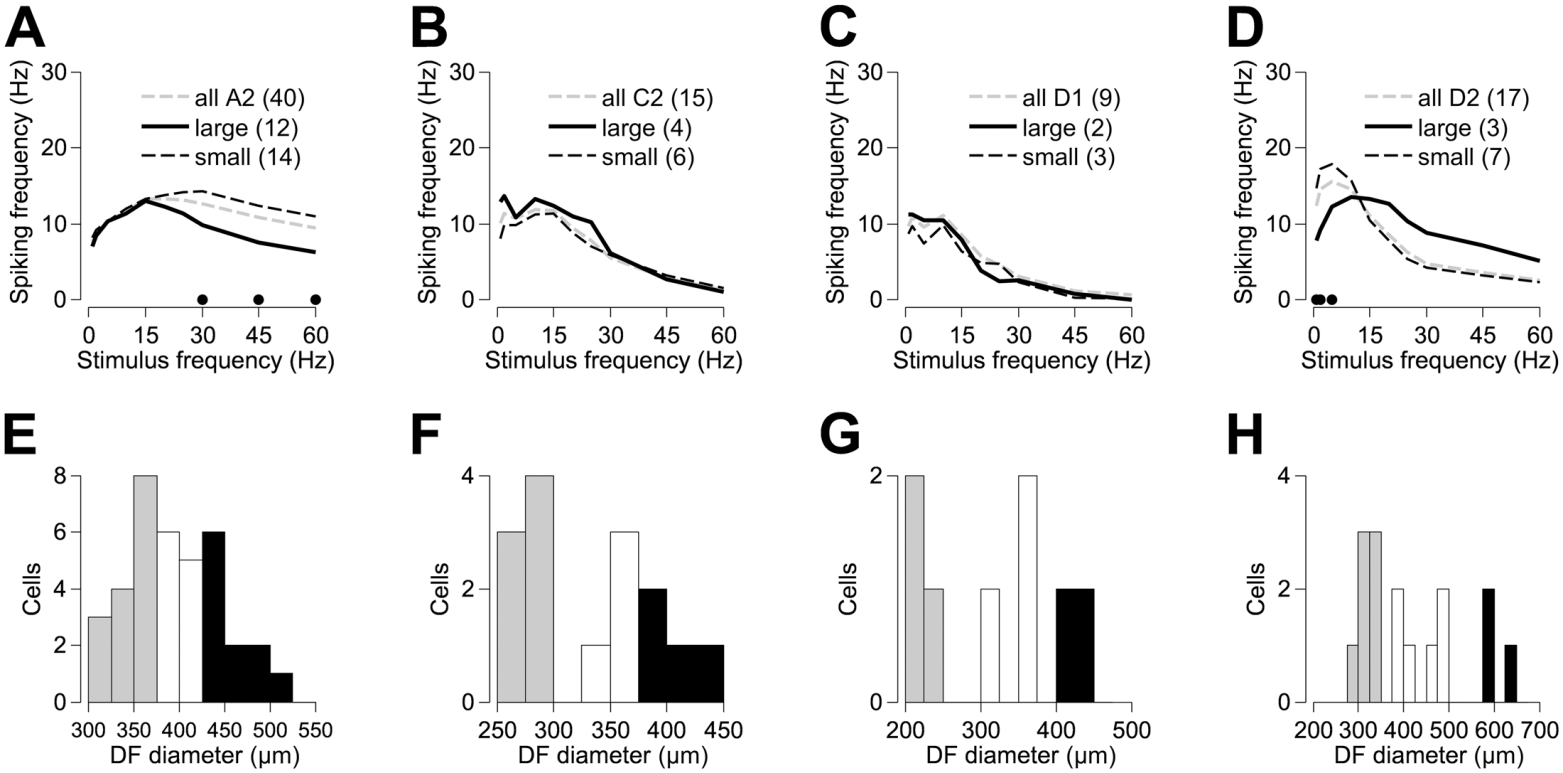
The effect of dendritic field size on the frequency response of retinal ganglion cells. Panels (A–D) show the mean frequency response of A2, C2, D1 and D2 RGC types. Within each RGC type, cells are grouped and their frequency responses averaged according to their dendritic field diameter. Within each RGC type, cells with the largest dendritic fields are shown in black and those with the smallest dendritic fields are shown in grey. For comparison, the frequency response, averaged over all cells of a given type, irrespective of dendritic field size, is shown by the dashed line. Black dots indicate statistically significant differences between large-field and small-field RGC responses (t-tests, p < 0.05). Panels (E–H) show distributions of dendritic field diameter for each of the A2, C2, D1 and D2 RGC types, respectively.

The effect of dendritic field size was found to vary with cell type. Fig 7A–7D show frequency responses for cells with large and small dendritic field diameters within each morphological cell type. Fig 7E–7H show distributions of dendritic field diameter for each cell type, indicating those cells in the upper and lower tails of each distribution designated as large (black bars; {A2, C2, D1, D2 dendritic field diameter} > {420, 370, 400, 500 μm}) and small (grey bars; {A2, C2, D1, D2 dendritic field diameter} < {370, 290, 300, 350 μm}) cells, respectively. A2 cells with large dendritic field diameters (>420 μm) were not as responsive to high frequency stimuli (p < 0.05 for frequencies ≥30 Hz) as those with small dendritic field diameters (<375 μm). Large-field D2 cells (>575 μm) were also outperformed by their small-field counterparts (<350 μm) when excited by low frequency stimuli (p < 0.049 for frequencies ≤5 Hz), although their average spiking response was greater when stimulated by higher frequencies (p > 0.282 for frequencies ≥20 Hz). Dendritic field size was also found to influence the mean spike probability (see Fig 4) in these two cell classes. In response to 60 Hz stimuli, the spiking probability for small-field A2 cells converged to ~17%, with large-field A2 cells converging to ~9%. Similarly, spiking probability in large- and small-field D2 cells reached ~8% and ~3%, respectively (data not shown). Large-field and small-field cells of the C2 and D1 classes had similar frequency responses (C2: p > 0.085; D1: p > 0.423).

We found an insignificant negative correlation between threshold current and soma diameter (Pearson's linear correlation coefficient, ρ = -0.195, p = 0.070), and no correlation between threshold and dendritic field diameter (ρ = 0.017, p = 0.870).

### Intrinsic physiological properties

As seen in Fig 3, the frequency responses of RGCs can vary even within a given morphological cell type. How much of this variation can be attributed to variation in the cells’ intrinsic physiological properties? We investigated this in a sub-set of our recorded RGCs by correlating their observed frequency response with direct measurements of their intrinsic physiological properties (Table 1). To estimate the cells’ intrinsic properties we recorded the membrane potential whilst subjecting the recorded cell to a series of brief (400 ms duration) hyperpolarizing and depolarizing constant current pulses [5]. We then computed: (1) resting membrane potential, V_rest_, (2) maximum firing frequency, f_max_, (3) steady-state firing frequency, f_ss_, (4), frequency adaptation index, FA, (5) spike width, sw, (6) input resistance, R_n_, and (7) sag (anomalous rectification in response to hyperpolarizing current). Sag was quantified by measuring the positive shift in membrane potential towards V_rest_ when the peak hyperpolarization reached -85 mV. Maximum firing rate (f_max_) and steady-state firing rate (f_ss_) were calculated using the average interspike interval of the first and last three spikes elicited, respectively, during a 400 ms depolarizing current pulse. The frequency adaptation (FA) index was calculated with the formula:

$$FA=\frac{f_{max}-f_{ss}}{f_{max}}.$$ (2)

**Table 1:** Intrinsic Physiological Properties of RGC Subtypes.

Type	n	V_rest_ (mV)	f_max_ (Hz)	f_ss_ (Hz)	FA	SW (ms)	R_N_ (MΩ)	sag (mV)
A1	3	-61 (3)	88 (25)	39 (14)	0.56 (0.07)	2.34 (0.09)	181 (18)	-11.8 (3)
A2i	9	-59 (6)	99 (24)	36 (12)	0.64 (0.09)	1.27 (0.43)	72 (19)	-0.9 (0.1)
A2o	31	-61 (5)	148 (46)	53 (14)	0.63 (0.11)	1.01 (0.24)	113 (48)	-2.3 (2.5)
C2i	4	-59 (3)	123 (23)	44 (18)	0.65 (0.09)	1.3 (0.33)	283 (14)	-6.5 (1.2)
C2o	11	-65 (7)	116 (50)	47 (21)	0.58 (0.12)	1.3 (0.33)	321 (203)	-3 (2.5)
C3	2	-66 (6)	73 (2)	33 (3)	0.54 (0.06)	1.91 (0.04)	144 *	-7.3 *
C4o	5	-58 (6)	112 (57)	44 (20)	0.58 (0.13)	1.53 (0.11)	379 (123)	-0.6 (1.3)
D1	9	-61 (9)	90 (52)	37 (13)	0.52 (0.12)	2.09 (0.74)	271 (132)	-11.4 (2.2)
D2	17	-63 (5)	110 (42)	46 (22)	0.58 (0.1)	1.61 (0.52)	193 (47)	-7.2 (6.1)

V_rest_, resting membrane potential; f_max_, maximum firing rate; f_ss_, steady-state firing rate; FA, frequency adaptation index; SW, spike width; R_N_, input resistance; sag, rectification of membrane potential back toward resting level in response to hyperpolarization. Values are means (standard deviation).

*indicates data from a single cell.

As a control, we compared our estimates of these parameters with the mean values for rat RGC types, as reported previously [5]. We found no significant difference between the reported intrinsic properties for rat RGCs and those of our sample, except for the D1 cell type. For this cell type our sample exhibited a lower mean value for sag (-11.1 mV) compared to that reported previously (-1.7 mV).

Of the intrinsic and morphological properties we measured, no single property could adequately account for the observed variation in frequency response (i.e., r^2^ < 0.2 over all stimulus frequencies). We then assessed the extent to which a combination of these properties could account for the observed frequency responses to sinusoidal stimulus currents using multiple linear regression. At low stimulation frequencies (<10 Hz) the cells’ intrinsic properties appear to play very little role in determining their frequency response: i.e. no combination of intrinsic and morphological properties could explain the variation in frequency response over this range of stimulus frequencies. However, variations in spike width, input resistance, and sag account for an increasing proportion of the variance observed in the cells’ frequency response with greater stimulus frequencies, explaining 37–38% of the total response variation for stimulus frequencies of 45 Hz and 60 Hz. This percentage fell to 29% for stimulation at 30 Hz, and was further reduced for lower stimulus frequencies. Soma diameter was found to be highly correlated with input resistance (Pearson's linear correlation coefficient, ρ = -0.587, p < 0.001), spike width (ρ = -0.426, p < 0.001), and sag (ρ = 0.395, p = 0.003), and could account for 41% of the total response variation for stimulation at 60 Hz, with this percentage falling to 33% and 18% for stimulation at 45 Hz and 30 Hz, respectively.

## Discussion

We have shown that morphological RGC types in rat retina differ in their frequency responses when assessed using intracellular sinusoidal current injection. In general, RGCs were less responsive to stimuli with higher frequencies, and this effect was heavily dependent on morphological cell type (Fig 3). Accordingly, all cells either had low-pass or band-pass frequency responses. Several cell types were notable for their response characteristics. A2-type cells, characterised by their large somata and dendritic fields, were most responsive to higher stimulus frequencies, cutting off at 56–68 Hz. At lower stimulus frequencies, the A2 cells were outperformed by the C1 and C4o cell types, which were highly responsive to stimuli of 15–30 Hz. These cells responded to almost every cycle delivered at 15 Hz, in stark contrast to all other cell types. The results presented here can be compared directly to those measured from cat alpha and beta cells [13]. In cat the two retinal cell types have similar band-pass frequency tuning characteristics, which compare reasonably well to rat A, B2 and B3 cell types.

In addition to the variation across cell types, we found some variability within each morphological class. A high level of variability in the responses to extracellular sinusoidal stimulation within the functional ON, OFF, and ON-OFF classes of rabbit RGCs has been reported, although cells did not receive anatomical classification in that study [22]. The rabbit retina hosts a minimum of 13 different RGC types, which includes ON and OFF variants within the morphological classifications [23]. Comparing our data from the rat, in which we have assessed the morphology and stratification of a sizeable population of cells, with that from the rabbit [23], it is not surprising that the rabbit study observed such a high degree of variability in response to sinusoidal stimulation.

### Influence of stratification and intrinsic properties

Our results show that classification into ON and OFF cells (based on stratification within the inner plexiform layer) does not reveal any significant differences in frequency tuning within either of the A2 or C2 types, although OFF A2-cells (i.e. A2o) appear to be less receptive to intracellular sinusoidal stimuli than ON A2-cells. We also measured intrinsic physiological properties for each cell to assess their influence on the observed frequency responses. Our analysis determined that the influence of three parameters in particular (spike width, input resistance, and sag) increased with stimulus frequency, explaining more than a third of the total response variation for frequencies above 30 Hz.

### Influence of cell size

We observed that dendritic field size had a differential effect on two cell types: the A2 and D2 classes. Small-field A2 cells were more responsive to high frequency (≥30 Hz) stimulation, and large-field D2 cells were more responsive to low frequency (≤5 Hz) stimulation, while being less receptive to stimulation at higher frequencies. Stimulus thresholds have been found to be inversely proportional to dendritic field size in RGCs recorded from explanted rabbit retinal preparations [24]. In a previous study, we investigated the ability of the alpha-type rat A2 RGC in sustaining high frequency extracellular stimulation [25]. While we found a weak trend for cells with small dendritic fields to have higher median thresholds, cells with smaller somas and/or dendritic fields were better able to keep up with repetitive stimulation at frequencies of 10–200 Hz, with the advantage increasing with stimulus frequency. These results from extracellular stimulation differ from the intracellular stimulation data presented here, possibly indicating the influence of presynaptic neural networks.

Soma size, after controlling for cell type, was found to be inconsequential in the ability of RGCs to respond to repetitive intracellular stimulation, although on average, cells with large somas outperformed those with smaller somas. Our findings confirm that cells with lower stimulus thresholds can better sustain repetitive activation at high frequencies (≥30 Hz), and that these cells are more likely to have large somas. This agrees with *in vitro* studies of the rabbit retina, where it was found that the lowest thresholds belonged to the alpha-like brisk transient RGCs, with smaller-soma types having the largest thresholds [26]. Cho et al. [27] investigated the relationship between soma size and threshold in the wild-type mouse (C57BL/6) and also found smaller thresholds in larger RGCs. This result seems intuitive, as the number of conducting ion channels (and therefore cell excitability) should increase as a function of total membrane surface area, provided that ion channel density is conserved. While the relationship between soma diameter and threshold current does not reach significance in our study, the weak negative correlation lends support to this argument.

In light of the response variation we observe within each morphological cell type, our results suggest that other factors (aside from cell size, stratification, and the intrinsic cellular properties of input resistance, sag, and spike width) have a role in determining the ability of a RGC to respond to intracellular sinusoidal stimulation. Previous work in rabbit determined the most excitable region of the RGC membrane to be the sodium channel band, a dense expression of voltage-gated sodium channels located within the axon initial segment (AIS) that varies in size and location across different functional RGC types [26]. The rat AIS is known to host several different voltage-gated ion channel populations, including the Na_v_1.6 subtype, whose presence allows for sustained firing during repetitive stimulation at high frequency [28,29]. In other neural systems diversity in ion channel expression, even within a single cell type, has been shown to have considerable effects on spiking properties. Measurements of potassium ion channel (voltage-gated- and large-conductance calcium-activated) expression in lateral pyloric and pyloric dilator cells of the crab have shown that there can be considerable variation within cells of a single type, translating into a diverse range of spiking outputs [30]. The same study deduced that cells with similar spiking properties could alternatively express differing proportions of these ion channels. Günay et al. [31] have used models generated from recordings of globus pallidus (GP) neurons in Sprague Dawley rat brain slices to demonstrate that the substantial variation of spike rate and overall excitability in GP neurons can be attributed to variations in voltage-gated ion channel density, and that the effect of an individual ion channel population is dependent on the presence of other ion channel subtypes. We suggest that the precise distributions of these ion channels underlie the ability of different RGCs to respond to both intracellular and extracellular repetitive stimulation.

### Adaptation

During visual stimulation, most neurons throughout the visual system adapt, i.e. for a given steady-state visual input the response decays over time (e.g. retina: [32,33]; lateral geniculate nucleus: [34]; accessory optic system: [35]; visual cortex: [36,37]). A rapid form of adaptation in the retina adjusts sensitivity and shortens the impulse responses of retinal ganglion cells in just 100 ms (e.g. [33,38]). Slower adaptation also occurs over many seconds and adjusts responsiveness to the overall contrast level of the scene [33]. In RGCs slow adaptation is associated with hyperpolarization of the membrane potential [33].

Based on single cell recording alone, it is difficult to establish if these stimulus-driven changes in response gain to a steady visual input are driven by presynaptic neural networks, the intrinsic properties of the neurons or both. In the present paradigm we injected current intracellularly, so any indication of response decay over time (adaptation) is likely to relate to intrinsic membrane characteristics. All cell types generated spikes at stimulus onset but in all but the C1 and C2o cells (at 15 Hz), the ability to fire spikes beyond the first few cycles declined. For most cells the reduction in efficacy occurred very rapidly, usually within 3–4 cycles of current injection. These results suggest that other intrinsic properties of the cells (aside from those investigated in our study) should exhibit substantial adaptive influences on the firing properties of the neurons during visual stimulation.

Several mechanisms are possibly recruited by adaptation. Much evidence points towards the role of pre-synaptic networks in driving adaptation, e.g. synaptic depression due to the depletion of vesicles from the presynaptic terminal [39] or the change in the strength of synaptic inhibition [40,41]. Given the use of intracellular injection in our study, we will not discuss the pre-synaptic components. What is known of intrinsic influences? Sodium channel inactivation contributes to adaptation-related changes in contrast sensitivity in the retina [42]. The time scale of recovery from inactivation of sodium channels correlates with the time period of the depolarization [43]. Intracellular recordings from cortex have shown that adaptation causes little change in synaptic input but strong after-hyperpolarization [44]. The latter is likely derived from the activation of sodium-gated potassium channels associated with the influx of sodium generated by both synaptic input and the generation of action potentials [45,46]. Importantly in the present context, the injection of steady-state depolarizing current also activates these channels and creates a drop in spike rates over time that resembles what occurs during visual adaptation. The distribution of voltage-gated sodium and potassium channels on the AIS is known to depend on cell type, and is associated with functional differences [26,47,48]. Given the clear changes in spiking frequency over time observed in our data, it is highly likely that the expression of voltage-gated ion channels influences responses to any steady input (either through electrical or visual stimulation), and may also determine the type-specific differences in response decay observed in our study.
